# Effect of Stiffness on Reflection and Transmission of Waves at an Interface between Heat Conducting Elastic Solid and Micropolar Fluid Media

**DOI:** 10.1155/2014/312840

**Published:** 2014-10-29

**Authors:** Rajneesh Kumar, S. C. Rajvanshi, Mandeep Kaur

**Affiliations:** ^1^Department of Mathematics, Kurukshetra University, Kurukshetra 136119, India; ^2^Research Centre, Punjab Technical University, Kapurthala, India; ^3^Department of Applied Sciences, Gurukul Vidyapeeth Institute of Engineering and Technology, Banur, Sector 7, Patiala District, Punjab 140601, India

## Abstract

The present investigation is concerned with the propagation of waves at an imperfect boundary of heat conducting elastic solid and micropolar fluid media. The amplitude ratios of various reflected and transmitted waves are obtained in a closed form due to incidence of longitudinal wave (P-wave), thermal wave (T-wave), and transverse wave (SV-wave). The variation of various amplitude ratios with angle of incidence is obtained for normal force stiffness, transverse force stiffness, thermal contact conductance, and perfect bonding. Numerical results are shown graphically to depict the effect of stiffness and thermal relaxation times on resulting quantities. Some particular cases are also deduced in the present investigation.

## 1. Introduction

The fluids in which coupling between the spin of each fluid particle and the microscopic velocity field is taken into account are termed as micropolar fluids. They represent fluids consisting of rigid, randomly oriented, or spherical particles suspended in a viscous medium, where the deformation of fluid particles is ignored. The theory of microfluids was introduced by Eringen [1]. A microfluid possesses three gyration vector fields in addition to its classical translatory degrees of freedom. As a subclass of these fluids, Eringen introduced the micropolar fluids [2] to describe the physical systems, which do not fall in the realm of viscous fluids. In micropolar fluids the local fluid elements were allowed to undergo only rigid rotations without stretch. These fluids support couple stress, the body couples, and asymmetric stress tensor and possess a rotational field, which is independent of the velocity of fluid. A large class of fluids such as anisotropic fluids, liquid crystals with rigid molecules, magnetic fluids, cloud with dust, muddy fluids, biological fluids, and dirty fluids (dusty air, snow) can be modeled more realistically as micropolar fluids. The importance of micropolar fluids in industrial applications has motivated many researchers to extend the study in numerous ways to explain various physical effects.

Ariman et al. [3, 4] studied microcontinuum fluid mechanics and its applications and Říha [5] investigated the theory of heat-conducting micropolar fluid with microtemperature. Eringen and Kafadar [6] discussed polar field theories, Brulin [7] studied linear micropolar media, Gorla [8] investigated combined forced and free convection in the boundary layer flow of a micropolar fluid on a continuous moving vertical cylinder, and Eringen [9] studied the theory of thermo-microstretch fluids and bubbly liquid. Aydemir and Venart [10] investigated flow of a thermomicropolar fluid with stretch. Ciarletta [11] established two sorts of spatial decay estimates for describing the spatial behaviour of the solutions for the flow of a heat-conducting micropolar fluid in a semi-infinite cylindrical pipe. The lateral surface is taken to be thermal insulated and the adherence of the fluid to the lateral boundary is assumed. A time-dependent velocity and angular velocity profile are prescribed together with the temperature field at the finite end of the pipe. Hsia and Cheng [12] studied longitudinal plane waves propagation in elastic micropolar porous media. Propagation of transverse waves in elastic micropolar porous semispaces is discussed by Hsia et al. [13].

Different researchers studied the problems of reflection and transmission of plane waves at an interface of micropolar elastic half-spaces (Tomar and Gogna [14–16] and Kumar et al. [17, 18]). Singh and Tomar [19] discussed the longitudinal waves at an interface of micropolar fluid/micropolar solid half-spaces. Sun et al. [20] studied propagation characteristics of longitudinal displacement wave in micropolar fluid with micropolar elastic plate. Xu et al. [21] discussed reflection and transmission characteristics of coupled wave through micropolar elastic solid interlayer in micropolar fluid. Dang et al. [22] investigated propagation characteristics of coupled wave through micropolar fluid interlayer in micropolar elastic solid. Fu and Wei [23] investigated wave propagation through the imperfect interface between two micropolar solids. Sharma and Marin [24] studied reflection and transmission of waves from imperfect boundary between two heat conducting micropolar thermoelastic solids. Vishwakarma et al. [25] studied torsional wave propagation in an earth's crustal layer under the influence of imperfect interface.

The purpose of the present study is to study the problem of reflection and transmission of plane waves at an imperfect interface between heat conducting elastic solid and micropolar fluid media. Effects of stiffness and thermal relaxation times on the amplitude ratios for incidence of various plane waves, for example, longitudinal wave (P-wave), thermal wave (T-wave), and transverse wave (SV-wave), are depicted numerically and shown graphically with angle of incidence.

## 2. Basic Equations

The field equations in a homogeneous and isotropic elastic medium in the context of generalized theories of thermoelasticity, without body forces and heat sources, are given by Lord and Shulman [26] and Green and Lindsay [27] as

(1) (λ+2μ)∇(∇·u→)−μ(∇×(∇×u→))  −ν(1+τ1∂∂t)∇T=ρ∂2u→∂t2,K∗ΔT=ρc∗(∂T∂t+τ0∂2T∂t2)+νT0(∂∂t+η0τ0∂2∂t2)(∇·u→),

and the constitutive relations are

(2) tij=λur,rδij+μ(ui,j+uj,i)−ν(T+τ1∂T∂t)δij,hhhhhhhhhhhhhhhhhhhhhhhhhi,j,r=1,2,3,

where *ν* = (3*λ* + 2*μ*)*α*_*T*_ and the meaning of symbols is defined in the list at the end of the paper. The thermal relaxation times *τ*_0_ and *τ*_1_ satisfy the inequalities *τ*_0_ ≥ *τ*_1_ ≥ 0 for G-L theory only and Δ = ∂^2^/∂*x*_1_^2^ + ∂^2^/∂*x*_3_^2^ is the Laplacian operator. For Lord Shulman (L-S) theory *η*_0_ = 1, *τ*_1_ = 0 and for Green-Lindsay (G-L) theory *η*_0_ = 0, *τ*_1_ > 0.

Following Ciarletta [11], the field equations and the constitutive relations for heat conducting micropolar fluids without body forces, body couples, and heat sources are given by

(3) D1v→+(λf+μf)∇(∇·v→)+Kf(∇×Ψ→)−b∇Tf  −c0∇ϕ∗f=0,D2Ψ→+(αf+βf)∇(∇·Ψ→)+Kf(∇×v→)=0,

(4) $$\begin{array}{c} K_{1}^{*}\Delta T^{f}-bT_{0}^{f}\left(\nabla \cdot \vec{v}\right)=\rho^{f}aT_{0}^{f}\frac{\partial T^{f}}{\partial t}, \end{array}$$

(5) $$\begin{array}{c} \rho^{f}\frac{\partial \phi^{*f}}{\partial t}=\nabla \cdot \vec{v}, \end{array}$$

where

(6) $$\begin{array}{c} D_{1}=\left(\mu^{f}+K^{f}\right)\Delta -\rho^{f}\frac{\partial }{\partial t},\;\;D_{2}=\gamma^{f}\Delta -I\frac{\partial }{\partial t}-2K^{f}, \end{array}$$

such that superscript *f* denotes physical quantities and material constants related to the fluid and the constitutive relations are

(7) $$\begin{array}{c} t_{ij}^{f}=-p\delta_{ij}+\sigma_{ij}^{f},\\ p=bT^{f}+c_{0}\phi^{*f},\\ \sigma_{ij}^{f}=\lambda^{f}\gamma_{rr}\delta_{ij}+\left(\mu^{f}+K^{f}\right)\gamma_{ij}+\mu^{f}\gamma_{ji},\\ m_{ij}^{f}=\alpha^{f}\upsilon_{rr}\delta_{ij}+\beta^{f}\upsilon_{ji}+\gamma^{f}\upsilon_{ij}, \end{array}$$

where *γ*_*ij*_ = *v*_*j*,*i*_ + *ε*_*jir*_Ψ_*r*_, *υ*_*ij*_ = Ψ_*j*,*i*_, *b* = (3*λ* ^*f*^ + 2*μ* ^*f*^ + *K* ^*f*^)*α*_*T*^*f*^_ and symbols are defined in the list at the end of the paper.

## 3. Formulation of the Problem

**Figure 1:**
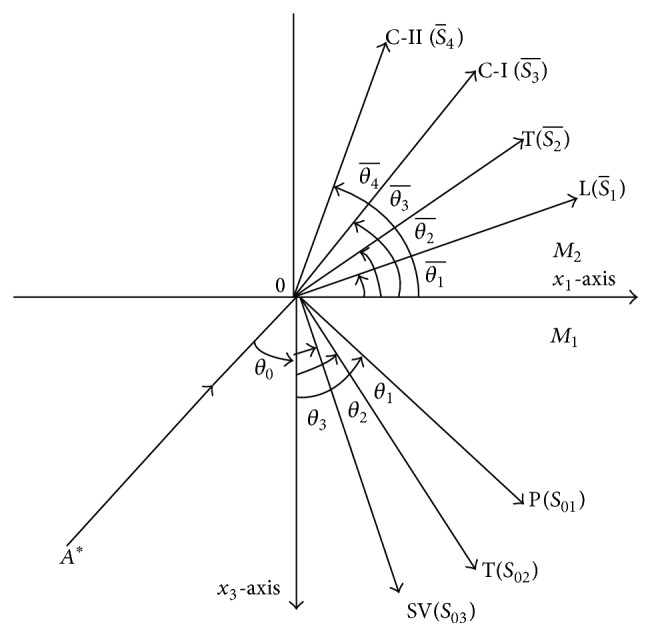
Geometry of the problem.

An imperfect interface of a homogeneous, isotropic generalized thermoelastic half-space (medium *M*_1_) in contact with heat conducting micropolar fluid half-space (medium *M*_2_) is considered. The rectangular Cartesian coordinate system O*x*_1_ *x*_2_ *x*_3_ having origin on the surface *x*_3_ = 0 seperating the two media is taken. Let us take the *x*_1_-axis along the interface between two half-spaces, namely, *M*_1_ (0 < *x*_3_ < *∞*) and *M*_2_ (−*∞* < *x*_3_ < 0), in such a way that *x*_3_-axis is pointing vertically downward into the medium *M*_1_. The geometry of the problem is shown in Figure 1.

For two dimensional problem in *x*_1_ *x*_3_-plane, we take the displacement vector $\vec{u}$, velocity vector $\vec{v}$, and microrotation velocity vector $\vec{\Psi }$ as

(8) $$\begin{array}{c} \vec{u}=\left(u_{1}\left(x_{1},x_{3}\right),0,u_{3}\left(x_{1},x_{3}\right)\right),\\ \vec{v}=\left(v_{1}\left(x_{1},x_{3}\right),0,v_{3}\left(x_{1},x_{3}\right)\right),\;\;\vec{\Psi }=\left(0,\Psi_{2}\left(x_{1},x_{3}\right),0\right). \end{array}$$

The following nondimensional quantities are defined:

(9) xi′=ω∗xic1,  ui′=ρω∗c1νT0ui,  vi′=ρc1νT0vi,ψ2′=ρc12ω∗νT0ψ2,  (t′,τ0′,τ1′)=(ω∗t,ω∗τ0,ω∗τ1),(T′,Tf′)=(TT0,TfT0),  (tij′,tijf′)=1νT0(tij,tijf),mijf′=ω∗c1νT0mijf,  ϕ∗f′=ρϕ∗f,Kn′=c1νT0Kn,  Kt′=c1νT0Kt,  Kθ′=1νc1Kθ,

where *ω* ^*^ = *ρc* ^*^ *c*_1_^2^/*K* ^*^, *c*_1_^2^ = (*λ* + 2*μ*)/*ρ*.

The displacement components *u*_1_, *u*_3_ and velocity components *v*_1_, *v*_3_ are related to the potential functions *ϕ*, *ϕ* ^*f*^ and *ψ*, *ψ* ^*f*^ in dimensionless form as

(10) $$\begin{array}{c} \left(u_{1},v_{1}\right)=\left(\frac{\partial }{\partial x_{1}}\left(\phi ,\phi^{f}\right)-\frac{\partial }{\partial x_{3}}\left(\psi ,\psi^{f}\right)\right),\\ \left(u_{3},v_{3}\right)=\left(\frac{\partial }{\partial x_{3}}\left(\phi ,\phi^{f}\right)+\frac{\partial }{\partial x_{1}}\left(\psi ,\psi^{f}\right)\right). \end{array}$$

Using (10) in (1) and (3)–(5) and with the aid of (8) and (9) (after suppressing the primes), we obtain

(11) $$\begin{array}{c} \nabla^{2}\phi -\left(1+\tau_{1}\frac{\partial }{\partial t}\right)T-\frac{\partial^{2}\phi }{\partial t^{2}}=0,\\ \nabla^{2}\psi -a_{1}\frac{\partial^{2}\psi }{\partial t^{2}}=0,\\ \nabla^{2}T=\left(1+\tau_{0}\frac{\partial }{\partial t}\right)\frac{\partial T}{\partial t}+\varepsilon_{1}\left(\frac{\partial }{\partial t}+\eta_{0}\tau_{0}\frac{\partial^{2}}{\partial t^{2}}\right)\nabla^{2}\phi ,\\ \nabla^{2}\phi^{f}-b_{1}T^{f}-b_{2}\phi^{*f}-b_{3}\frac{\partial \phi^{f}}{\partial t}=0,\\ \nabla^{2}\psi^{f}+b_{4}\Psi_{2}-b_{5}\frac{\partial \psi^{f}}{\partial t}=0,\\ \nabla^{2}T^{f}-b_{9}\nabla^{2}\phi^{f}-b_{10}\frac{\partial T^{f}}{\partial t}=0,\\ \nabla^{2}\Psi_{2}-b_{6}\nabla^{2}\psi^{f}-b_{7}\Psi_{2}-b_{8}\frac{\partial \Psi_{2}}{\partial t}=0,\\ b_{11}\nabla^{2}\phi^{f}-\frac{\partial }{\partial t}\phi^{*f}=0, \end{array}$$

where

(12) $$\begin{array}{c} a_{1}=\frac{\rho c_{1}^{2}}{\mu },\;\;\varepsilon_{1}=\frac{\nu^{2}T_{0}}{K^{*}\omega^{*}\rho },\;\;b_{1}=\frac{b\rho c_{1}^{2}}{\left(\lambda^{f}+2\mu^{f}+K^{f}\right)\omega^{*}\nu },\\ b_{2}=\frac{c_{0}c_{1}^{2}}{\left(\lambda^{f}+2\mu^{f}+K^{f}\right)\omega^{*}\nu T_{0}},\\ b_{3}=\frac{\rho^{f}c_{1}^{2}}{\left(\lambda^{f}+2\mu^{f}+K^{f}\right)\omega^{*}},\;\;b_{4}=\frac{K^{f}}{\mu^{f}+K^{f}},\\ b_{5}=\frac{\rho^{f}c_{1}^{2}}{\left(\mu^{f}+K^{f}\right)\omega^{*}},\;\;b_{6}=\frac{K^{f}c_{1}^{2}}{\gamma^{f}\omega^{*2}},\\ b_{7}=2b_{6},\;\;b_{8}=\frac{Ic_{1}^{2}}{\gamma^{f}\omega^{*}},\;\;b_{9}=\frac{b\nu T_{0}^{f}}{K_{1}^{*}\rho \omega^{*}},\\ b_{10}=\frac{\rho^{f}aT_{0}^{f}c_{1}^{2}}{K_{1}^{*}\omega^{*}},\;\;b_{11}=\frac{\nu T_{0}}{\rho^{f}c_{1}^{2}}. \end{array}$$

## 4. Boundary Conditions

The boundary conditions at the interface *x*_3_ = 0 are defined as

(13) $$\begin{array}{c} t_{33}^{f}=K_{n}\left(\frac{\partial u_{3}}{\partial t}-v_{3}\right),\;\;t_{31}^{f}=K_{t}\left(\frac{\partial u_{1}}{\partial t}-v_{1}\right),\\ K_{1}^{*}\frac{\partial T^{f}}{\partial x_{3}}=K_{\theta }\left(T-T^{f}\right),\;\;t_{33}=t_{33}^{f},\;\;t_{31}=t_{31}^{f},\\ m_{32}^{f}=0,\;\;K^{*}\frac{\partial T}{\partial x_{3}}=K_{1}^{*}\frac{\partial T^{f}}{\partial x_{3}}, \end{array}$$

where *K*_*n*_, *K*_*t*_, and *K*_*θ*_ are the normal force stiffness, transverse force stiffness, and thermal contact conductance coefficients of unit layer thickness having dimensions N sec/m^3^, N sec/m^3^, and N/m sec K.

## 5. Reflection and Transmission

The longitudinal wave (P-wave) or thermal wave (T-wave) or transverse wave (SV-wave) propagating through the medium *M*_1_ which is designated as the region *x*_3_ > 0 is considered. The plane wave is taken to be incident at the plane *x*_3_ = 0 with its direction of propagation with angle *θ*_0_ normal to the surface. Each incident wave corresponds to reflected P-wave, T-wave, and SV-wave in medium *M*_1_ and transmitted longitudinal wave (L-wave), thermal wave (T-wave), and transverse longitudinal wave coupled with transverse microrotational wave (C-I and C-II waves) in medium *M*_2_ as shown in Figure 1.

In order to solve (11), we assume the solutions of the system of the form

(14) {ϕ,T,ψ,ϕf,ϕ∗f,Tf,ψf,Ψ2} ={ϕ¯,T¯,ψ¯,ϕf¯,ϕ∗f¯,Tf,¯ ψf¯,Ψ2¯}eι{k(x1sinθ−x3cos⁡θ)−ωt},

where *k* is the wave number, *ω* is the angular frequency, and $\bar{\phi }$, $\bar{T}$, $\bar{\psi }$, $\bar{\phi^{f}}$, $\bar{\phi^{*f}}$, $\bar{T^{f}}$, $\bar{\psi^{f}}$, $\bar{\Psi_{2}}$ are arbitrary constants.

Making use of (14) in (11), we obtain

(15) $$\begin{array}{c} V^{4}+D_{1}V^{2}+E_{1}=0, \end{array}$$

(16) $$\begin{array}{c} V^{4}+D_{2}V^{2}+E_{2}=0,\\ V^{4}+D_{3}V^{2}+E_{3}=0, \end{array}$$

where

(17) $$\begin{array}{c} D_{1}=-\frac{1}{\tau_{00}}\left[1+\left(1-\iota \tau_{1}\omega \right)\varepsilon_{1}\left(\frac{\iota }{\omega }+\eta_{0}\tau_{0}\right)\right]-1,\\ E_{1}=\frac{1}{\tau_{00}},\\ D_{2}=\frac{\iota \omega }{b_{3}}\left(1-\frac{\iota b_{2}b_{11}}{\omega }\right)+\frac{\iota \omega }{b_{10}}\left(1+\frac{\iota b_{1}b_{9}}{\omega b_{3}}\right),\\ E_{2}=\frac{-\omega^{2}}{b_{3}b_{10}}\left(1-\frac{\iota b_{2}b_{11}}{\omega }\right),\\ D_{3}=\frac{\iota \omega }{b_{5}}+\iota \omega \left[\frac{\left(1-\iota b_{4}b_{6}/\omega b_{5}\right)}{\left(b_{8}+\left(\iota /\omega \right)b_{7}\right)}\right],\\ E_{3}=-\frac{\omega^{2}}{\left(b_{8}+\left(\iota /\omega \right)b_{7}\right)b_{5}},\\ V^{2}=\frac{\omega^{2}}{k^{2}},\;\;\tau_{00}=\left(\frac{\iota }{\omega }+\tau_{0}\right). \end{array}$$

Here *V*_1_, *V*_2_ are the velocities of P-wave and T-wave in medium *M*_1_ and these are roots of (15) and $V_{3}=1/\sqrt{a_{1}}$ is the velocity of SV-wave in medium *M*_1_. $\bar{V_{1}}$, $\bar{V_{2}}$, $\bar{V_{3}}$, and $\bar{V_{4}}$ are the velocities of transmitted longitudinal wave (L-wave), thermal wave (T-wave), and transverse longitudinal wave coupled with microrotational wave (C-I and C-II) in medium *M*_2_. These are roots of (16).

In view of (14), the appropriate solutions of (11) for medium *M*_1_ and medium *M*_2_ are taken as follows.

Medium *M*_1_ is as follows:

(18) {ϕ,T}=∑i=12{1,fi}[S0ieι{ki(x1sinθ0i−x3cos⁡θ0i)−ωit}+Pi],ψ=S03eι{k3(x1sinθ03−x3cos⁡θ03)−ω3t}+S3eι{k3(x1sinθ03+x3cos⁡θ03)−ω3t},

where

(19) $$\begin{array}{c} f_{i}=\frac{\varepsilon_{1}\omega_{i}^{2}\left(\iota /\omega_{i}+\eta_{0}\tau_{0}\right)}{-1/V_{i}^{2}+\left(\iota /\omega_{i}+\tau_{0}\right)-\iota \varepsilon_{1}\omega_{i}\left(\iota /\omega_{i}+\eta_{0}\tau_{0}\right)\left(\iota /\omega_{i}+\tau_{1}\right)},\\ P_{i}=S_{i}e^{\iota \{k_{i}(x_{1}\sin\theta_{i}+x_{3}\cos\theta_{i})-\omega_{i}t\}}. \end{array}$$

Medium *M*_2_ is as follows:

(20) {ϕf,Tf,ϕ∗f} =∑i=12{1,fi¯,gi¯}Si¯eι{ki¯(x1sinθi¯−x3cos⁡θi¯)−ωi¯t},

(21) {ψf,Ψ2} =∑j=34{1,fj¯}Sj¯eι{kj¯(x1sinθj¯−x3cos⁡θj¯)−ωj¯t},

where

(22) $$\begin{array}{c} \bar{f_{i}}=\frac{-b_{3}b_{9}}{b_{1}b_{9}\left(\iota /\omega_{i}\right)+\iota \omega_{i}\left(1-\iota b_{2}b_{11}/\omega_{i}\right)\left(1/{\bar{V_{i}}}^{2}-b_{10}\left(\iota /\omega_{i}\right)\right)},\\ \bar{f_{j}}=\frac{\left(b_{6}b_{5}\left(\iota /\omega_{i}\right)\right)}{-1/{\bar{V_{j}}}^{2}-b_{7}/\omega_{j}^{2}+b_{8}\left(\iota /\omega_{i}\right)+b_{4}b_{6}/\omega_{j}^{2}},\;\;\bar{g_{i}}=\frac{-\iota b_{11}\omega_{i}}{{\bar{V_{i}}}^{2}}, \end{array}$$

and *S*_0*i*_, *S*_03_ are the amplitudes of incident (P-wave, T-wave) and SV-wave, respectively. *S*_*i*_ and *S*_3_ are the amplitudes of reflected (P-wave, T-wave) and SV-wave and $\bar{S_{i}}$, $\bar{S_{j}}$ are the amplitudes of transmitted longitudinal wave, thermal wave, and transverse longitudinal wave coupled with transverse microrotational wave, respectively.

Equation (20) represents the relation between (*ϕ* ^*f*^ and *T* ^*f*^) and (*ϕ* ^*f*^ and *ϕ* ^*f*∗^).

Snell's law is given by

(23) sinθ0V0=sinθ1V1=sinθ2V2=sinθ3V3=sinθ1¯V1¯=sinθ2¯V2¯=sinθ3¯V3¯=sinθ4¯V4¯,

where

(24) k1V1=k2V2=k3V3=k1¯ V1¯=k2¯ V2¯=k3¯ V3¯=k4¯ V4¯=ω,hhhhhhhhhhhhhhhhhhhhhhhhhhhhhhhhhhhat  x3=0.

Making use of (18)–(21) in the boundary conditions (13) and with the help of (2), (7), (9), (10), (23), and (24), we obtain a system of seven nonhomogeneous equations which can be written as

(25) $$\begin{array}{c} \overset{7}{\underset{j=1}{\sum }}a_{ij}Z_{j}=Y_{i};\;\left(i=1,2,3,4,5,6,7\right), \end{array}$$

where the values of *a*_*ij*_ are given in the Appendix.

(1) For incident P-wave,
  (26) A∗=S01,  S02=S03=0,  Y1=a11,Y2=−a21,  Y3=−a31,  Y4=−a41,Y5=a51,  Y6=0,  Y7=a71.
(2) For incident T-wave,
  (27) A∗=S02,  S01=S03=0,  Y1=a12,Y2=−a22,  Y3=−a32,  Y4=−a42,Y5=a52,  Y6=0,  Y7=a72.
(3) For incident SV-wave,
  (28) A∗=S03,  S01=S02=0,  Y1=−a13,Y2=a23,  Y3=a33=0,  Y4=−a43,Y5=−a53,  Y6=0,  Y7=a73=0,Z1=S1A∗,  Z2=S2A∗,  Z3=S3A∗,Z4=S1¯A∗, Z5=S2¯A∗, Z6=S3¯A∗, Z7=S4¯A∗,

where *Z*_1_, *Z*_2_, and *Z*_3_ are the amplitude ratios of reflected P-wave, T-wave, and SV-wave in medium *M*_1_ and *Z*_4_, *Z*_5_, *Z*_6_, and *Z*_7_ are the amplitude ratios of transmitted longitudinal wave (L-wave), thermal wave (T-wave), and transverse longitudinal wave coupled with transverse microrotational wave (C-I and C-II waves) in medium *M*_2_.

## 6. Particular Cases

### 6.1. Case I: Normal Force Stiffness

*K*_*n*_ ≠ 0, *K*_*t*_ → *∞*, and *K*_*θ*_ → *∞* in (25) yield the resulting quantities for normal force stiffness and lead a system of seven nonhomogeneous equations given by (A.1) with the changed values of *a*_*ij*_ as

(29) $$\begin{array}{c} a_{2i}=-\frac{\omega^{2}}{V_{0}}\sin\theta_{0},\;\;a_{23}=\frac{\omega^{2}}{V_{3}}\sqrt{1-\frac{V_{3}^{2}}{V_{0}^{2}}\sin^{2}\theta_{0}},\\ a_{24}=\iota \frac{\omega }{V_{0}}\sin\theta_{0},\;\;a_{25}=\iota \frac{\omega }{V_{0}}\sin\theta_{0},\\ a_{26}=\iota \frac{\omega }{\bar{V_{3}}}\sqrt{1-\frac{\bar{V_{3}^{2}}}{V_{0}^{2}}\sin^{2}\theta_{0}},\;\;a_{27}=\iota \frac{\omega }{\bar{V_{4}}}\sqrt{1-\frac{\bar{V_{4}^{2}}}{V_{0}^{2}}\sin^{2}\theta_{0}},\\ a_{3i}=f_{i},\;\;a_{33}=0,\;\;a_{34}=-\bar{f_{1}},\\ a_{35}=-\bar{f_{2}},\;\;a_{36}=a_{37}=0. \end{array}$$

### 6.2. Case II: Transverse Force Stiffness

*K*_*t*_ ≠ 0, *K*_*n*_ → *∞*, and *K*_*θ*_ → *∞* in (25) provide the case of transverse force stiffness giving a system of seven nonhomogeneous equations given by (A.1) with the changed values of *a*_*ij*_ as

(30) $$\begin{array}{c} a_{1i}=-\frac{\omega^{2}}{V_{i}}\sqrt{1-\frac{V_{i}^{2}}{V_{0}^{2}}\sin^{2}\theta_{0}},\\ a_{13}=-\frac{\omega^{2}}{V_{0}}\sin\theta_{0},\;\;a_{14}=-\left\{\iota \frac{\omega }{\bar{V_{1}}}\left(\sqrt{1-\frac{\bar{V_{1}^{2}}}{V_{0}^{2}}\sin^{2}\theta_{0}}\right)\right\},\\ a_{15}=-\left\{\iota \frac{\omega }{\bar{V_{2}}}\left(\sqrt{1-\frac{\bar{V_{2}^{2}}}{V_{0}^{2}}\sin^{2}\theta_{0}}\right)\right\},\;\;a_{16}=\iota \frac{\omega }{V_{0}}\sin\theta_{0},\\ a_{17}=\iota \frac{\omega }{V_{0}}\sin\theta_{0},\;\;a_{3i}=f_{i},\;\;a_{33}=0,\\ a_{34}=-\bar{f_{1}},\;\;a_{35}=-\bar{f_{2}},\;\;a_{36}=a_{37}=0. \end{array}$$

### 6.3. Case III: Thermal Contact Conductance

Taking *K*_*θ*_ ≠ 0, *K*_*t*_ → *∞*, and *K*_*n*_ → *∞* in (25) corresponds to the case of thermal contact conductance and yields a system of seven nonhomogeneous equations as given by (A.1) with the changed values of *a*_*ij*_ as

(31) a1i=−ω2Vi1−Vi2V02sin2θ0,  a13=−ω2V0sinθ0,a14=−{ιωV1¯(1−V12¯V02sin2θ0)},a15=−{ιωV2¯(1−V22¯V02sin2θ0)},a16=a17=ιωV0sinθ0,  a2i=−ω2V0sinθ0,a23=ω2V31−V32V02sin2θ0,  a24=ιωV0sinθ0,a25=ιωV0sinθ0,  a26=ιωV3¯1−V32¯V02sin2θ0,a27=ιωV4¯1−V42¯V02sin2θ0.

### 6.4. Case IV: Perfect Bonding

If we take *K*_*n*_ → *∞*, *K*_*t*_ → *∞*, and *K*_*θ*_ → *∞* in (25), we obtain the case of perfect bonding and yield a system of seven nonhomogeneous equations as given by (A.1) with the changed values of *a*_*ij*_ as

(32) $$\begin{array}{c} a_{1i}=-\frac{\omega^{2}}{V_{i}}\sqrt{1-\frac{V_{i}^{2}}{V_{0}^{2}}\sin^{2}\theta_{0}},\;\;a_{13}=-\frac{\omega^{2}}{V_{0}}\sin\theta_{0},\\ a_{14}=-\left\{\iota \frac{\omega }{\bar{V_{1}}}\left(\sqrt{1-\frac{\bar{V_{1}^{2}}}{V_{0}^{2}}\sin^{2}\theta_{0}}\right)\right\},\\ a_{15}=-\left\{\iota \frac{\omega }{\bar{V_{2}}}\left(\sqrt{1-\frac{\bar{V_{2}^{2}}}{V_{0}^{2}}\sin^{2}\theta_{0}}\right)\right\},\\ a_{16}=a_{17}=\iota \frac{\omega }{V_{0}}\sin\theta_{0},\;\;a_{2i}=-\frac{\omega^{2}}{V_{0}}\sin\theta_{0},\\ a_{23}=\frac{\omega^{2}}{V_{3}}\sqrt{1-\frac{V_{3}^{2}}{V_{0}^{2}}\sin^{2}\theta_{0}},\;\;a_{24}=\iota \frac{\omega }{V_{0}}\sin\theta_{0},\\ a_{25}=\iota \frac{\omega }{V_{0}}\sin\theta_{0},\;\;a_{26}=\iota \frac{\omega }{\bar{V_{3}}}\sqrt{1-\frac{\bar{V_{3}^{2}}}{V_{0}^{2}}\sin^{2}\theta_{0}},\\ a_{27}=\iota \frac{\omega }{\bar{V_{4}}}\sqrt{1-\frac{\bar{V_{4}^{2}}}{V_{0}^{2}}\sin^{2}\theta_{0}},\;\;a_{3i}=f_{i},\\ a_{33}=0,\;\;a_{34}=-\bar{f_{1}},\;\;a_{35}=-\bar{f_{2}},\;\;a_{36}=a_{37}=0. \end{array}$$

### 6.5. Subcases

(i) If micropolar heat conducting fluid medium is absent, we obtain the amplitude ratios at the free surface of thermoelastic solid with one relaxation time and two relaxation times.
 These results are similar to those obtained by A. N. Sinha and S. B. Sinha [28] for one relaxation time (L-S theory) and Sinha and Elsibai [29] for two relaxation times (G-L theory).
(ii) In the absence of upper medium *M*_2_, we obtain the amplitude ratios at the free surface of thermoelastic solid for CT-theory (*η*_0_ = *τ*_0_ = *τ*_1_ = 0).

These results are similar to those obtained by Deresiewicz [30] for CT-theory.

## 7. Special Cases

(i) If *η*_0_ = 1, *τ*_1_ = 0 in (25), (A.1), (29), (30), and (31) then we obtain the corresponding amplitude ratios at an interface of thermoelastic solid with one relaxation time and heat conducting micropolar fluid half-space for normal force stiffness, transverse force stiffness, and thermal contact conductance.
(ii) If *η*_0_ = 0, *τ*_1_ > 0 in (25), (A.1), (29), (30), and (31) then we obtain the corresponding amplitude ratios at an interface of thermoelastic solid with two relaxation times and heat conducting micropolar fluid half-space for normal force stiffness, transverse force stiffness, and thermal contact conductance.

## 8. Numerical Results and Discussion

The following values of relevant parameters for both the half-spaces for numerical computations are taken.

Following Singh and Tomar [19], the values of elastic constants for medium *M*_1_ are taken as

(33) $$\begin{array}{c} \lambda =0.209730\times 10^{10}\;\text{N}{\text{m}}^{-2},\;\;\mu =0.91822\times 10^{9}\;\text{N}{\text{m}}^{-2},\\ \rho =0.0034\times 10^{3}\;\text{Kg}\;{\text{m}}^{-3}, \end{array}$$

and thermal parameters are taken from Dhaliwal and Singh [31]:

(34) $$\begin{array}{c} \nu =0.268\times 10^{7}\;\text{N}{\text{m}}^{-2}\;{\text{K}}^{-1},\\ c^{*}=1.04\times 10^{3}\;\text{Nm}\;\text{K}{\text{g}}^{-1}\;{\text{K}}^{-1},\\ K^{*}=1.7\times 10^{2}\;\text{N}\;\text{se}{\text{c}}^{-1}\;{\text{K}}^{-1},\;\;T_{0}=0.298\;\text{K},\\ \tau_{0}=0.613\times 10^{-12}\;\text{sec},\;\;\tau_{1}=0.813\times 10^{-12}\;\text{sec},\\ \omega =1. \end{array}$$

Following Singh and Tomar [19], the values of micropolar constants for medium *M*_2_ are taken as

(35) $$\begin{array}{c} \lambda^{f}=1.5\times 10^{8}\;\text{N}\;\text{sec}\;{\text{m}}^{-2},\;\mu^{f}=0.03\times 10^{8}\;\text{N}\;\text{sec}\;{\text{m}}^{-2},\\ K^{f}=0.000223\times 10^{8}\;\text{N}\;\text{sec}\;{\text{m}}^{-2},\;\;\gamma^{f}=0.0000222\;\text{N}\;\text{sec},\\ \rho^{f}=0.8\times 10^{3}\;\text{Kg}\;{\text{m}}^{-3},\;\;I=0.00400\times 10^{-16}\;\text{N}\;{\text{sec}}^{2}\;{\text{m}}^{-2}. \end{array}$$

Thermal parameters for the medium *M*_2_ are taken as of comparable magnitude:

(36) $$\begin{array}{c} T_{0}^{f}=0.196\;\text{K},\;\;K_{1}^{*}=0.89\times 10^{2}\;\text{N}\;\text{se}{\text{c}}^{-1}\;{\text{K}}^{-1},\\ c_{0}=0.005\times 10^{11}\;\text{N}\;\text{se}{\text{c}}^{2}\;{\text{m}}^{-6},\\ a=1.5\times 10^{5}\;{\text{m}}^{2}\;\text{se}{\text{c}}^{-2}\;{\text{K}}^{-2},\;\;b=1.6\times 10^{5}\;\text{N}{\text{m}}^{-2}\;{\text{K}}^{-1}. \end{array}$$

The values of amplitude ratios have been computed at different angles of incidence.

In Figures 2 [Fig fig3] [Fig fig4] [Fig fig5] [Fig fig6] [Fig fig7] [Fig fig8] [Fig fig9] [Fig fig10] [Fig fig11] [Fig fig12] [Fig fig13] [Fig fig14] [Fig fig15] [Fig fig16] [Fig fig17] [Fig fig18] [Fig fig19] [Fig fig20] [Fig fig21]–22, for L-S theory, we represent the solid line for stiffness (ST1), small dashes line for normal force stiffness (NS1), medium dashes line for transverse force stiffness (TS1), and dash dot dash line for thermal contact conductance (TCS1). For G-L theory, we represent the dash double dot dash line for stiffness (ST2), solid line with center symbol “plus” for normal force stiffness (NS2), solid line with center symbol “diamond” for transverse force stiffness (TS2), and solid line with center symbol “cross” for thermal contact conductance (TCS2).

### 8.1. P-Wave Incident

**Figure 2:**
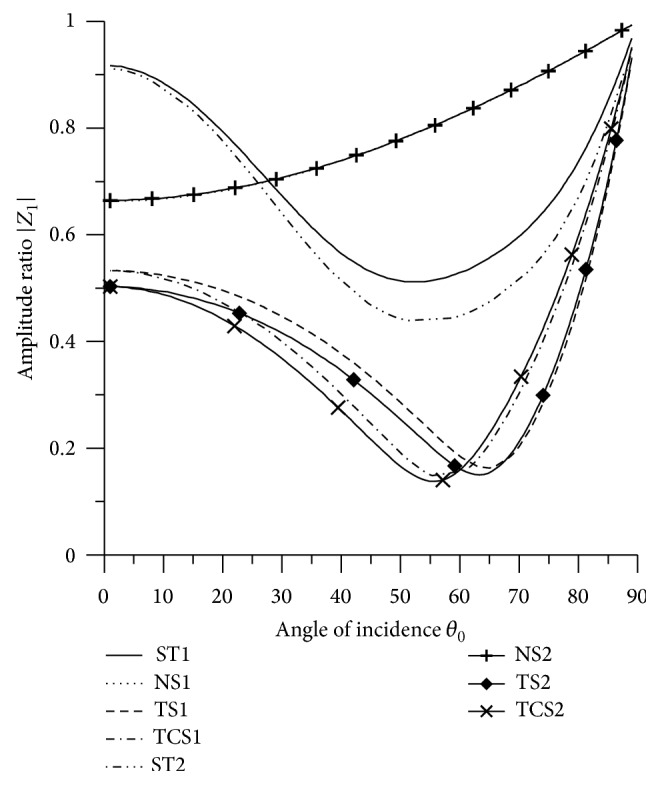
Variation of |*Z*_1_| with angle of incidence (P-wave).

**Figure 3 fig3:**
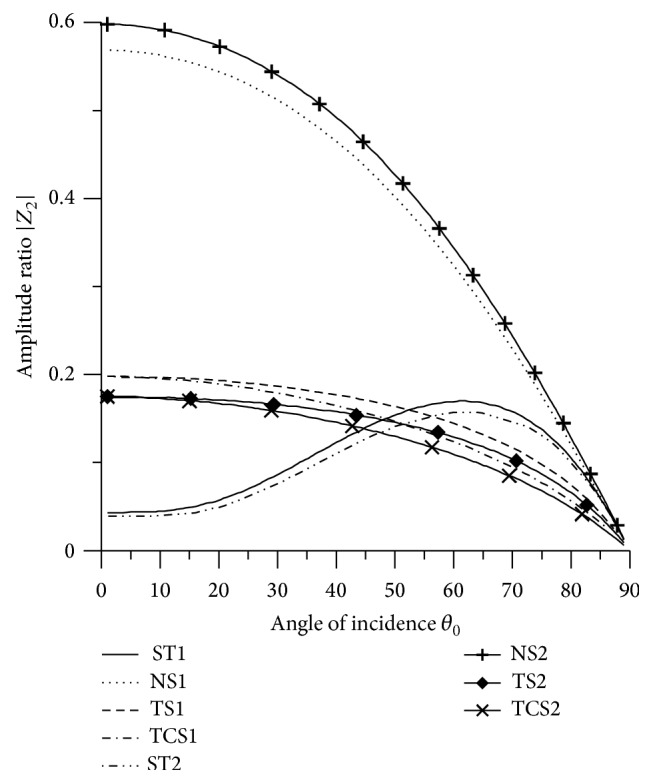
Variation of |*Z*_2_| with angle of incidence (P-wave).

**Figure 4 fig4:**
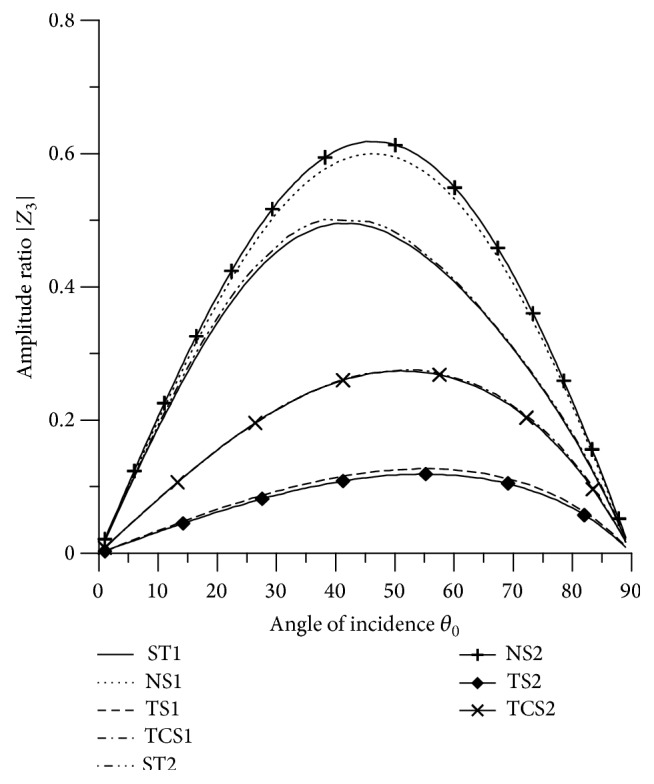
Variation of |*Z*_3_| with angle of incidence (P-wave).

**Figure 5 fig5:**
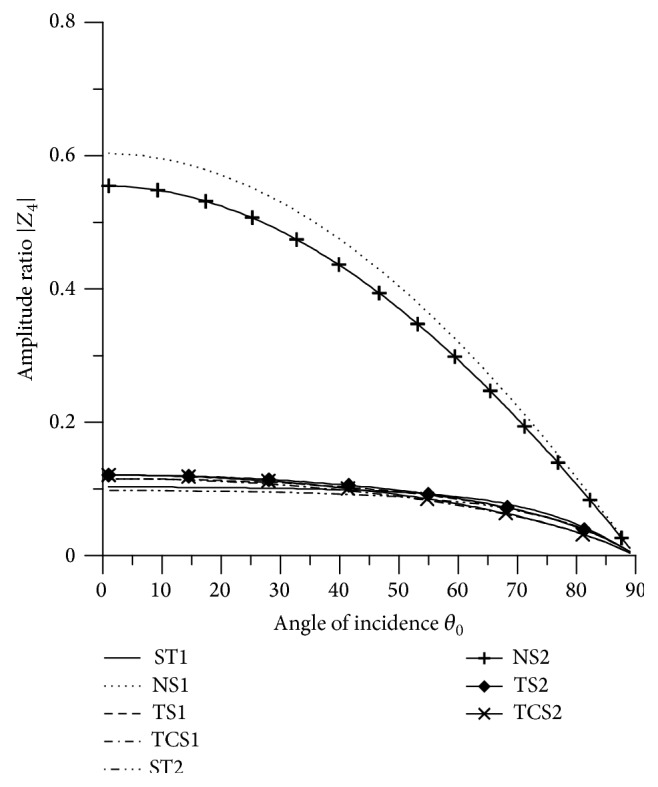
Variation of |*Z*_4_| with angle of incidence (P-wave).

**Figure 6 fig6:**
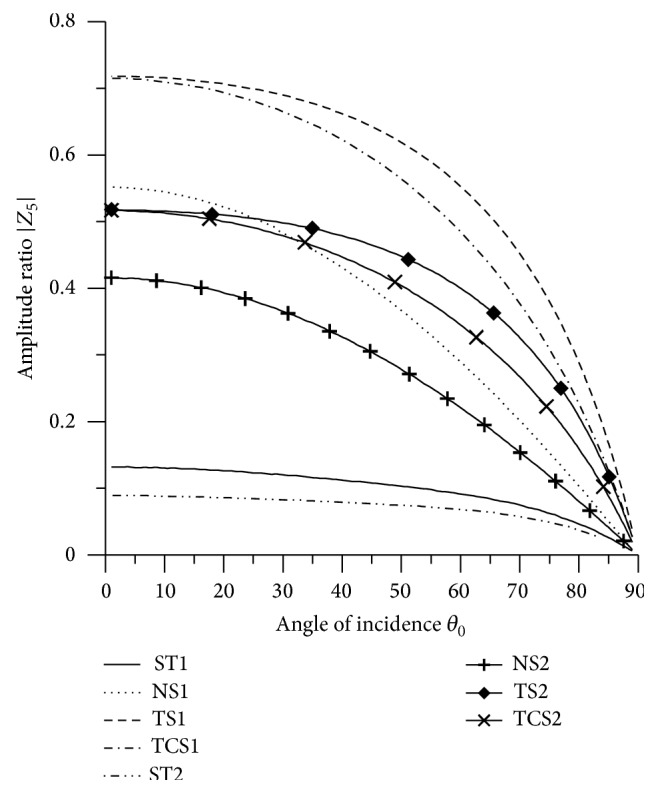
Variation of |*Z*_5_| with angle of incidence (P-wave).

**Figure 7 fig7:**
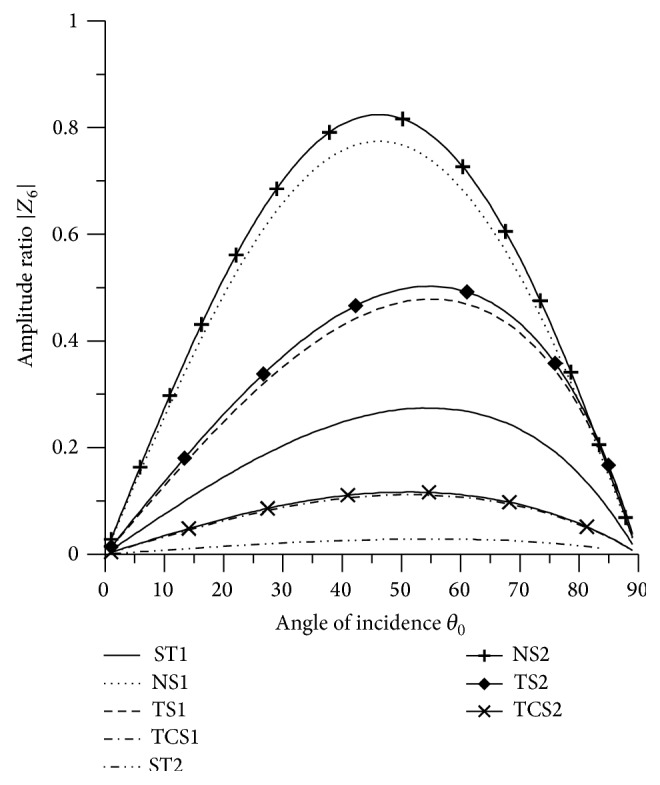
Variation of |*Z*_6_| with angle of incidence (P-wave).

**Figure 8 fig8:**
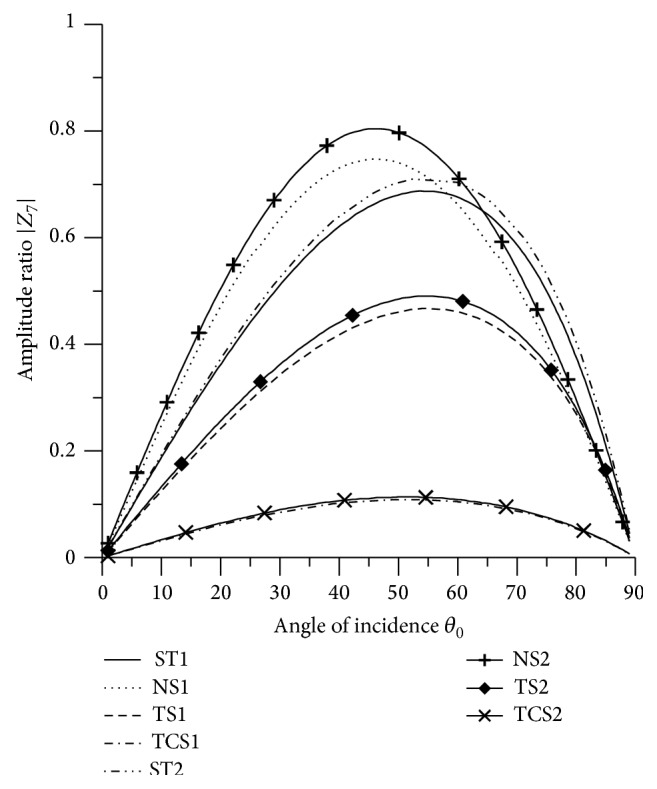
Variation of |*Z*_7_| with angle of incidence (P-wave).

Variations of amplitude ratios |*Z*_*i*_|, 1 ≤ *i* ≤ 7, with the angle of incidence *θ*_0_, for incident P-wave are shown in Figures 2 [Fig fig3] [Fig fig4] [Fig fig5] [Fig fig6] [Fig fig7]–8.

Figure 2 shows that the values of |*Z*_1_| for NS1 and NS2 increase in the whole range. The values for ST1, ST2, transverse force stiffness, and thermal contact conductance decrease in the whole range, except near the grazing incidence, where the values get increased. The values for ST1 remain more than the values for TS1, TS2, ST2, TCS1, and TCS2 in the whole range.

From Figure 3 it is evident that the values of |*Z*_2_| for all the stiffnesses, except ST1 and ST2, decrease in the whole range. The values of |*Z*_2_| for NS1 and NS2 are magnified by multiplying by 10.

Figure 4 shows that the values of |*Z*_3_| for all the stiffnesses for L-S theory and G-L theory first increase up to intermediate range and then decrease with the increase in *θ*_0_ and the values for ST2 are greater than the values for ST1, NS1, NS2, TS1, TS2, TCS1, and TCS2 in the whole range. The values of |*Z*_2_| for NS1 and NS2 are magnified by multiplying by 10.

From Figure 5 it is noticed that the values of |*Z*_4_| for all the stiffnesses start with maximum value at normal incidence and then decrease to attain minimum value at grazing incidence. The values of amplitude ratios for ST1 and NS1 are more than the values for ST2 and NS2, respectively, in the whole range. There is slight difference in the values of TCS1 and TCS2 in the whole range. The values of |*Z*_4_| for ST1, ST2, NS1, and NS2 are magnified by multiplying by 10^2^ and the values for TS1, TS2, TCS1, and TCS2 are magnified by multiplying by 10.

Figure 6 shows that the values of |*Z*_5_| for all the boundary stiffnesses decrease with increase in *θ* and the values of amplitude ratio for TS1 are greater than the values for all other boundary stiffnesses in the whole range that shows the effect of transverse force stiffness. The values of |*Z*_5_| for all the stiffnesses are magnified by multiplying by 10^2^.

From Figure 7 it is evident that the amplitude of |*Z*_6_| for normal force stiffness increases in the range 0° < *θ*_0_ < 48° and then decreases in the further range. The values for transverse force stiffness increase in the range 0° < *θ*_0_ < 59° and for thermal contact conductance increase in the range 0° < *θ*_0_ < 56° and then decrease. The values of |*Z*_6_| for all the stiffnesses, except TCS1 and TCS2, are magnified by multiplying by 10^3^, while the values for TCS1 and TCS2 are magnified by multiplying by 10^2^.

Figure 8 depicts that the values of |*Z*_7_| for ST1, ST2, TS1 and TS2 increase in the range 0° < *θ*_0_ < 56° and decrease in the remaining range. The values for ST2 and TS2 are greater than the values for ST1 and TS2 respectively in the whole range. The values for normal force stiffness for G-L theory remain more than the values for L-S theory. The values of |*Z*_7_| for ST1, ST2 are magnified by multiplying by 10^8^, the values for TCS1, TCS2 are magnified by multiplying by 10^6^ and the values for NS1, NS2, TS1 and TS2 are magnified by multiplying by 10^7^.

### 8.2. T-Wave Incident

**Figure 9 fig9:**
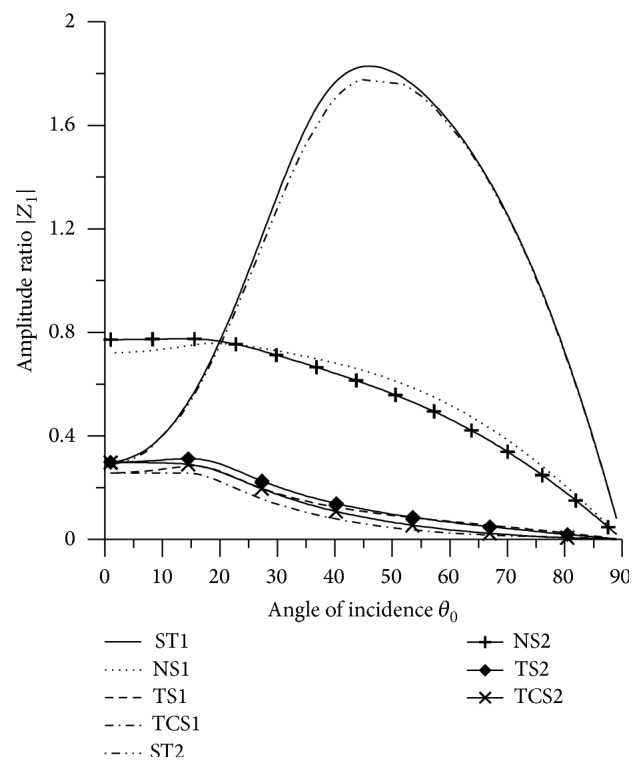
Variation of |*Z*_1_| with angle of incidence (P-wave).

The values of amplitude ratio |*Z*_1_| for transverse force stiffness and thermal contact conductance decrease in the whole range with slight increase in the initial range. The values for ST1 and ST2 increase in the range 0° < *θ*_0_ < 48° and then decrease. These variations have been shown in Figure 9. The values for TS1, TS2, TCS1 and TCS2 are reduced by dividing by 10.

**Figure 10 fig10:**
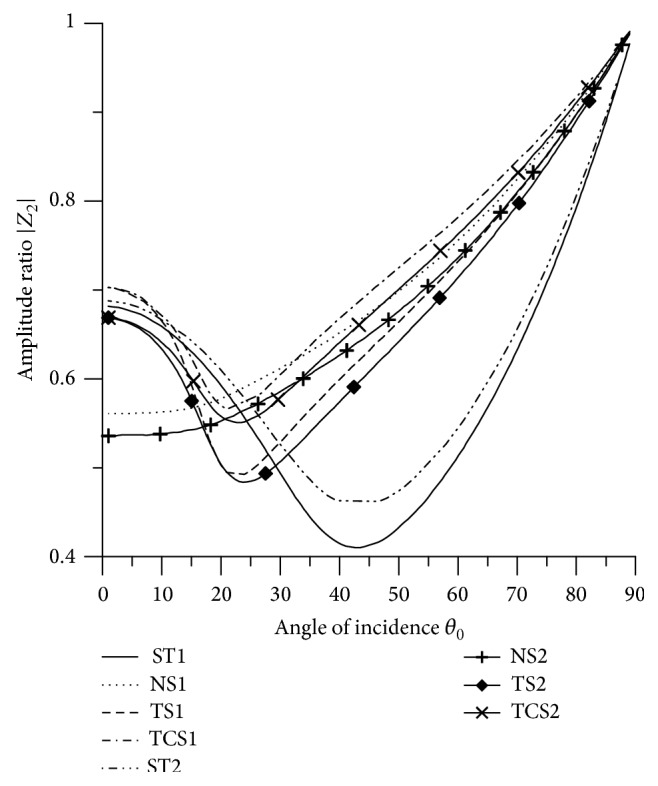
Variation of |*Z*_2_| with angle of incidence (P-wave).

The values of amplitude ratio |*Z*_2_| for NS1 and NS2 increase in the whole range, while the values for TS1, TS2, ST1, ST2, TCS1, TCS2 first decrease and then increase to attain maximum value at grazing incidence. These variations are shown in Figure 10.

**Figure 11 fig11:**
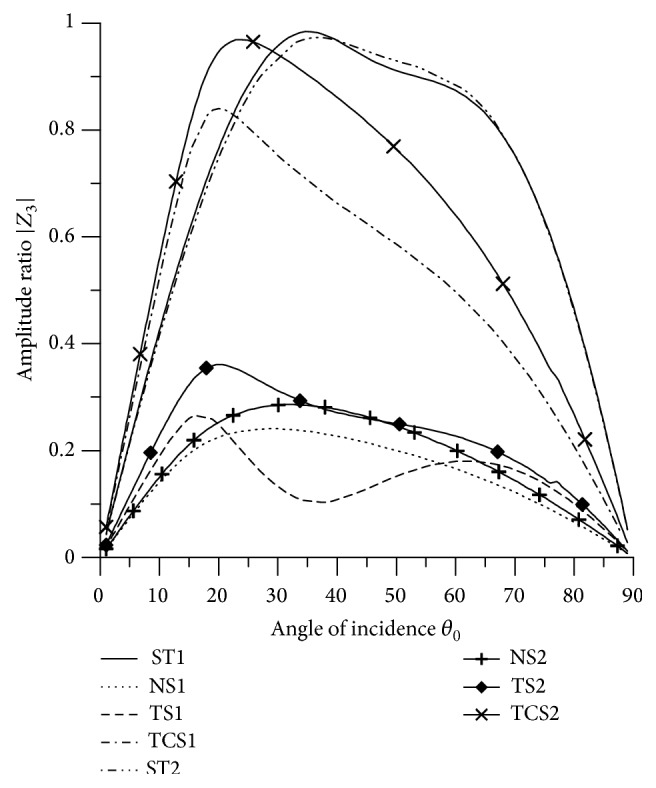
Variation of |*Z*_3_| with angle of incidence (T-wave).

From Figure 11, it is noticed that the values of |*Z*_3_| for normal force stiffness, transverse force stiffness, and thermal contact conductance for G-L theory are greater than the corresponding values for L-S theory. It is seen that the values for ST1 are greater than the values for ST2 in the range 0° < *θ*_0_ < 38° and, in the further range, the values for ST2 are more.

**Figure 12 fig12:**
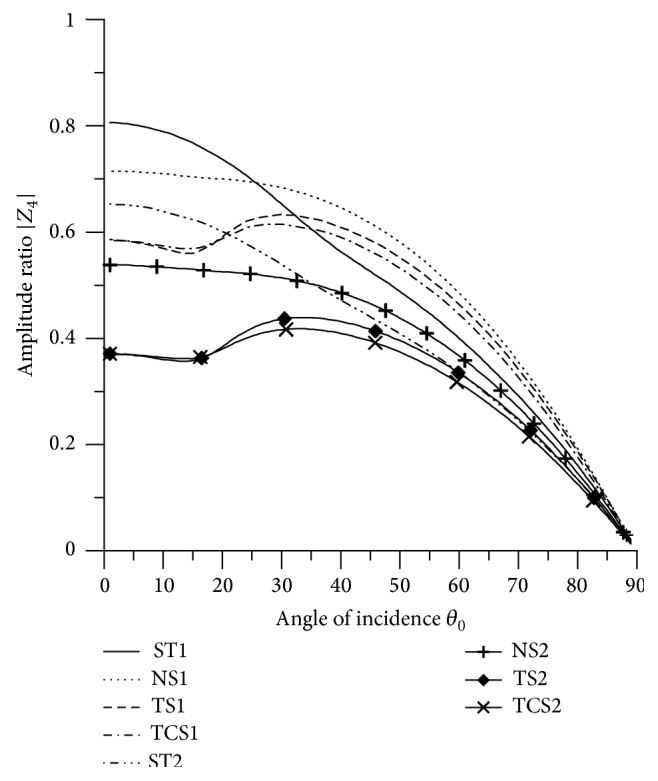
Variation of |*Z*_4_| with angle of incidence (T-wave).

Figure 12 depicts that the values of amplitude ratios |*Z*_4_| for transverse force stiffness and thermal contact conductance oscillate up to intermediate range and then decrease in the further range to attain minimum value at grazing incidence. The values for ST1, ST2, NS1, and NS2 decrease in the whole range. The values of |*Z*_4_| for ST1 and ST2 are magnified by multiplying by a factor of 10^2^ and NS1, NS2, TS1, TS2, TCS1, and TCS2 are magnified by a factor of 10.

**Figure 13 fig13:**
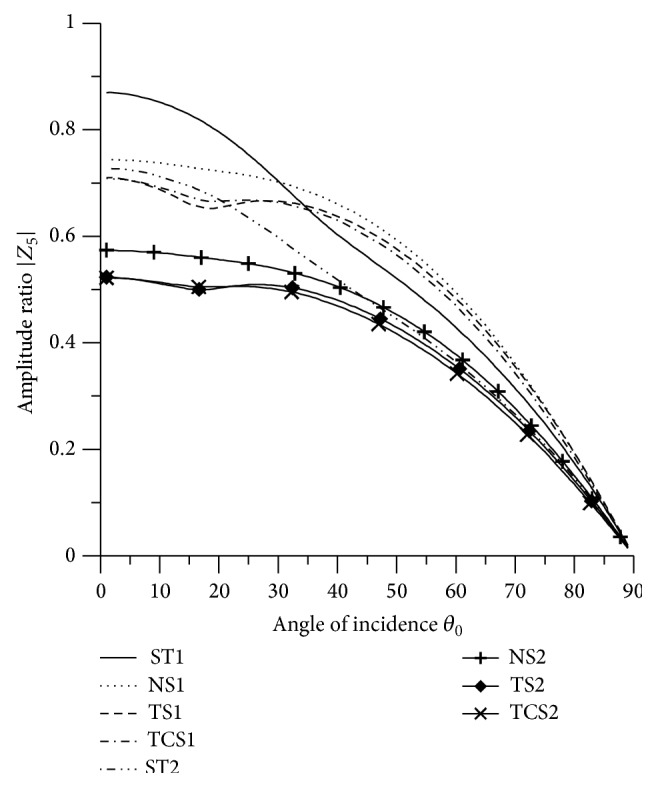
Variation of |*Z*_5_| with angle of incidence (T-wave).

Figure 13 shows that the behavior of variation of |*Z*_5_| for all the boundary stiffnesses is similar to that of |*Z*_4_|, but the magnitude of variation is different. The values of |*Z*_5_| for ST1 and ST2 are magnified by multiplying by a factor of 10^2^ and NS1, NS2, TS1, TS2, TCS1, and TCS2 are magnified by a factor of 10.

**Figure 14 fig14:**
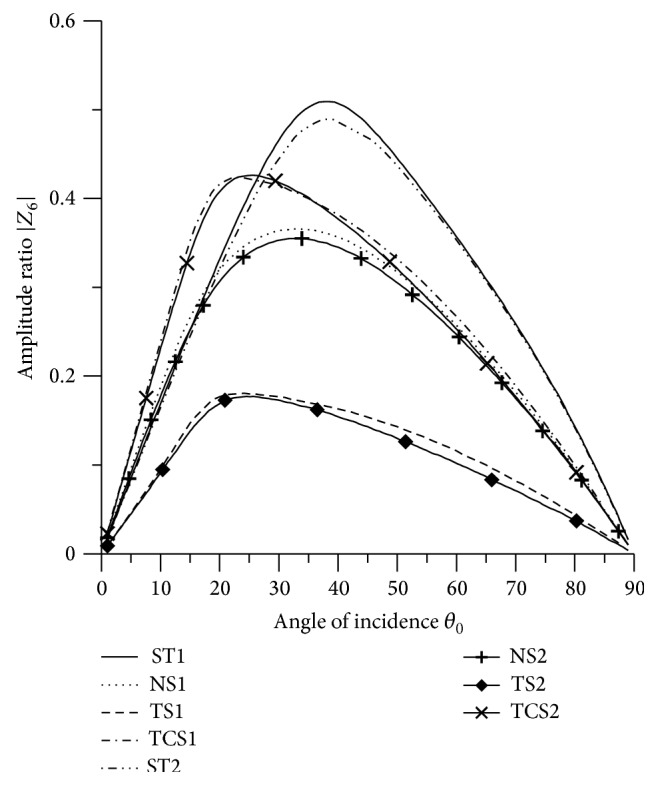
Variation of |*Z*_6_| with angle of incidence (T-wave).

From Figure 14 it is evident that the values of |*Z*_6_| for all the boundary stiffnesses increase to attain maximum value and then decrease up to grazing incidence. The values for NS1 and TS1 are greater than the values for NS2 and TS2, respectively, that reveals the thermal relaxation time effect. The values of |*Z*_5_| for ST1 and ST2 are magnified by multiplying by a factor of 10^3^ and NS1, NS2, TS1, TS2, TCS1, and TCS2 are magnified by a factor of 10^2^.

**Figure 15 fig15:**
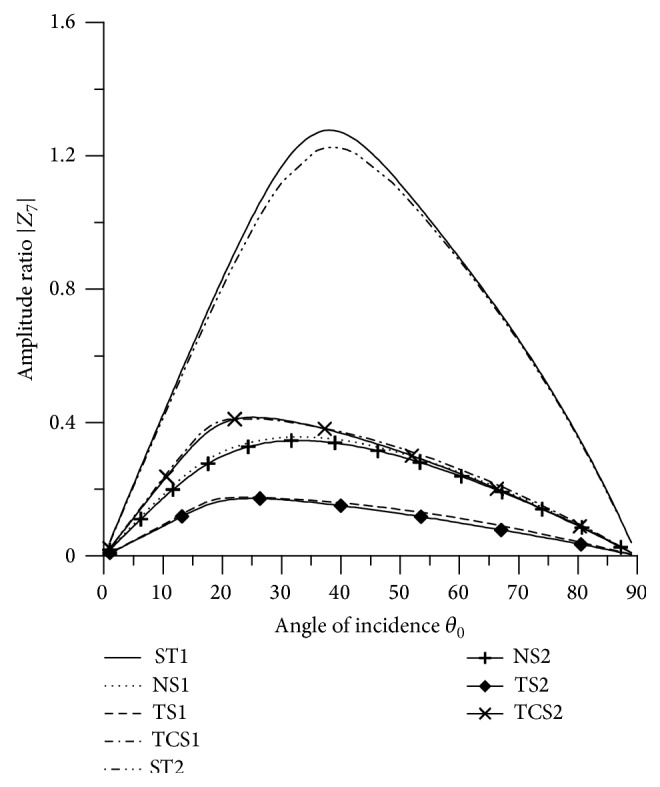
Variation of |*Z*_7_| with angle of incidence for T-wave.

Figure 15 depicts that the values of |*Z*_7_| for ST1 and ST2 increase in the interval 0° < *θ*_0_ < 39° and then decrease in the further range. It is noticed that there is slight difference in the values of amplitude ratio for L-S and G-L theory. The values of |*Z*_7_| for ST1 and ST2 are magnified by multiplying by a factor of 10^8^ and NS1, NS2, TS1, TS2, TCS1, and TCS2 are magnified by a factor of 10^6^.

### 8.3. SV Wave Incident

**Figure 16 fig16:**
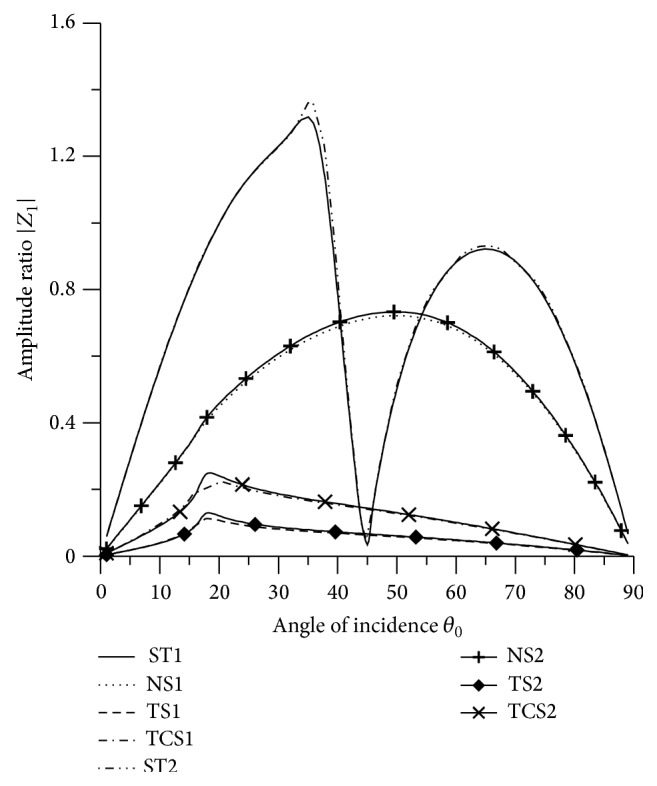
Variation of |*Z*_1_| with angle of incidence (SV-wave).

**Figure 17 fig17:**
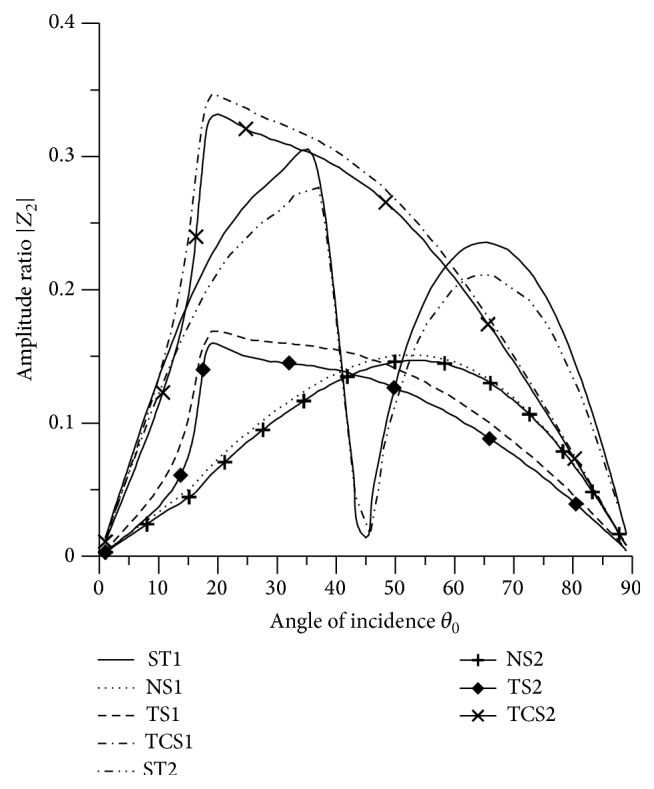
Variation of |*Z*_2_| with angle of incidence (SV-wave).

**Figure 18 fig18:**
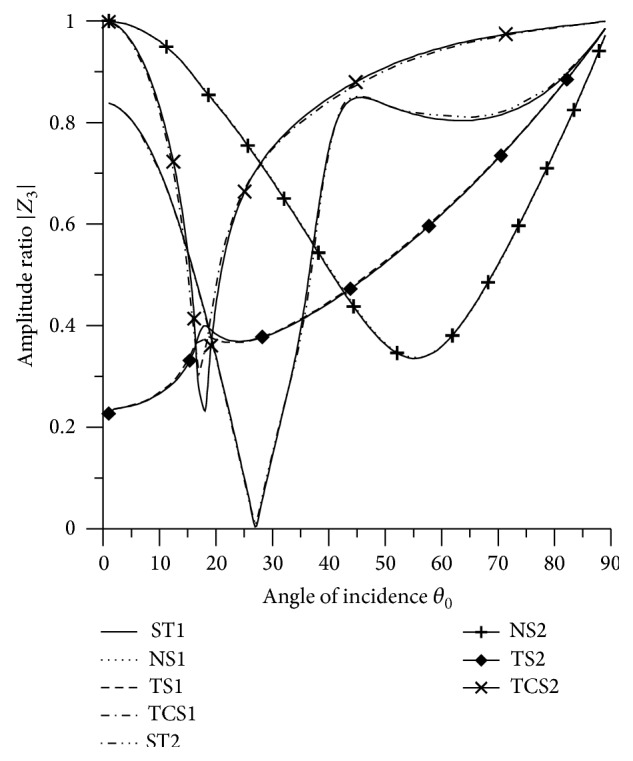
Variation of |*Z*_3_| with angle of incidence (SV-wave).

**Figure 19 fig19:**
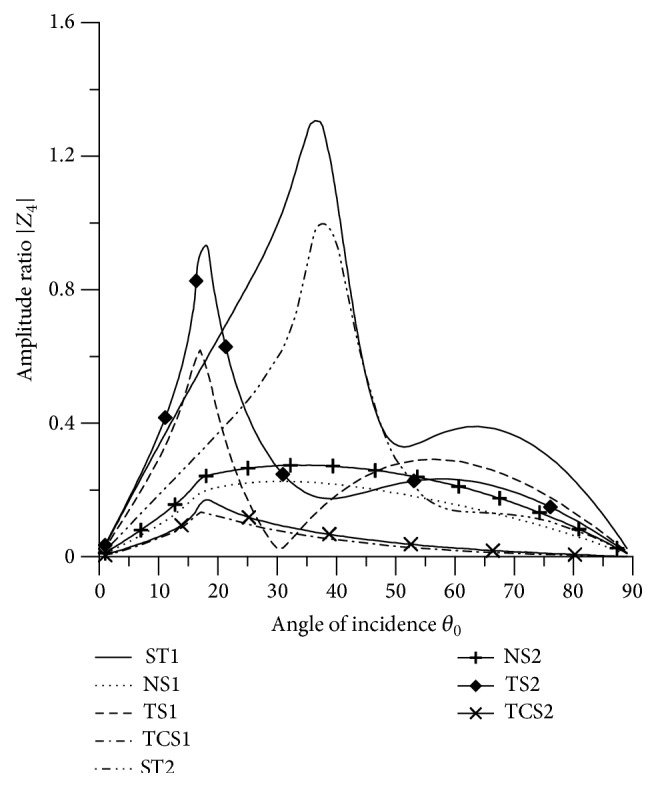
Variation of |*Z*_4_| with angle of incidence (SV-wave).

**Figure 20 fig20:**
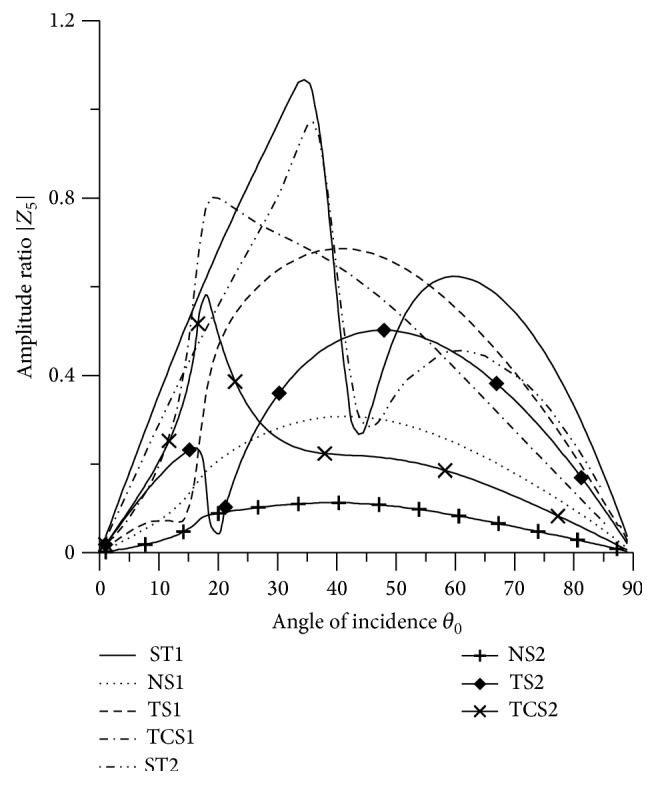
Variation of |*Z*_5_| with angle of incidence (SV-wave).

**Figure 21 fig21:**
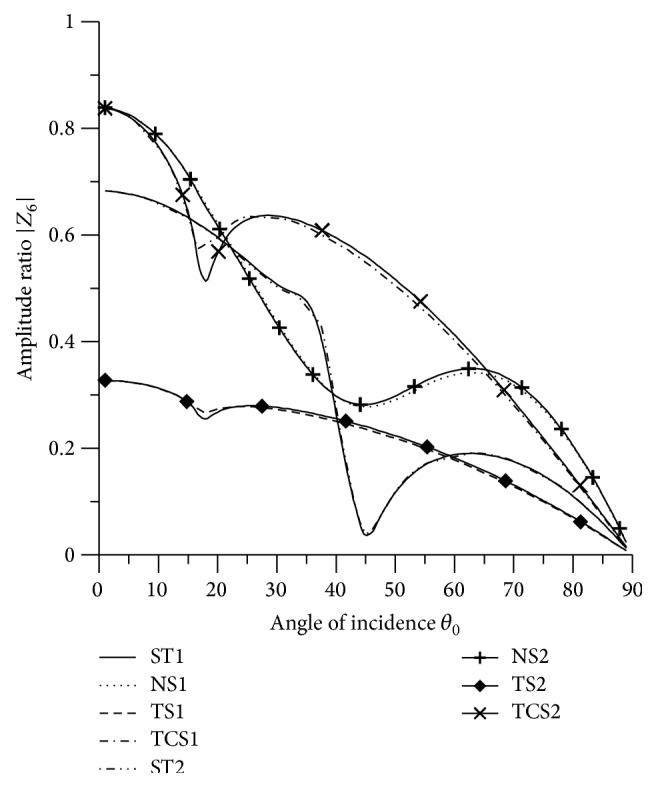
Variation of |*Z*_6_| with angle of incidence (SV-wave).

**Figure 22:**
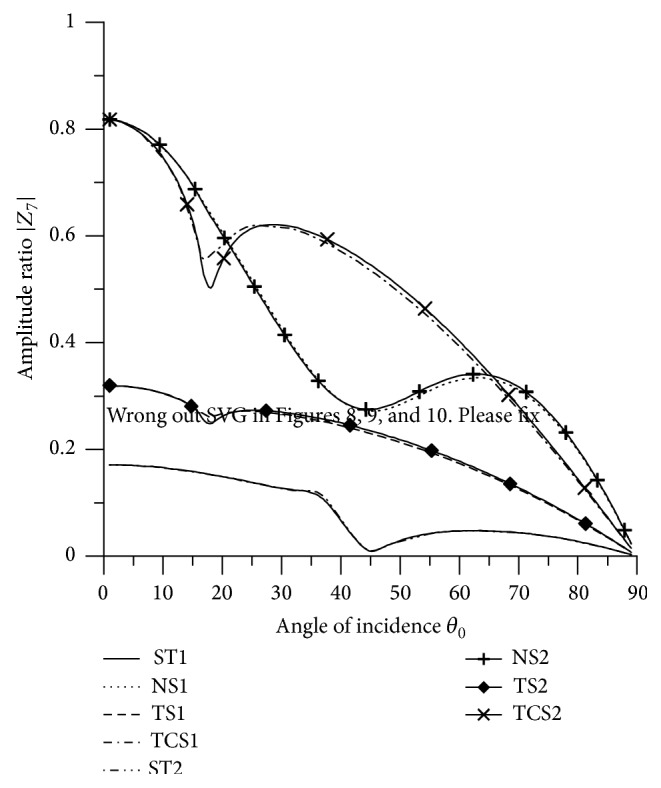
Variation of |*Z*_7_| with angle of incidence (SV-wave).

Variations of amplitude ratios |*Z*_*i*_|, 1 ≤ *i* ≤ 7, with the angle of incidence *θ*_0_, for incident SV-wave are shown in Figures 16 [Fig fig17] [Fig fig18] [Fig fig19] [Fig fig20] [Fig fig21]–22.

Figure 16 depicts that the values of |*Z*_1_| for transverse force stiffness and thermal contact conductance increase to attain peak values in the range 15° < *θ*_0_ < 25° and then decrease in the further range. The values for ST1 and ST2 increase in the range 0° < *θ*_0_ < 35° and 45° < *θ*_0_ < 66°, respectively, and decrease in the remaining range. The values for normal force stiffness increase from normal incidence to attain maximum value in the range 45° < *θ*_0_ < 55° and then decrease up to grazing incidence. The values for TS1, TS2, TCS1, and TCS2 are reduced by dividing by 10.

Figure 17 shows that values of amplitude ratio |*Z*_2_| for all the boundary stiffnesses follow oscillatory pattern in the whole range. The maximum value is attained by TCS1 in the range 15° < *θ*_0_ < 25°. It is seen that the values for L-S theory are greater than the values for G-L theory in the whole range.

Figure 18 shows that the values of |*Z*_3_| for transverse force stiffness increase from normal incidence to grazing incidence and attain peak value in the interval 15° < *θ*_0_ < 25°. The values for normal stiffness and thermal contact conductance decrease in the intervals 0° < *θ*_0_ < 56° and 0° < *θ*_0_ < 17°, respectively, and then increase in the further range.

Figure 19 shows that the values of |*Z*_4_| for all the boundary stiffnesses oscillate in the whole range. The values for ST1 are greater than the values for ST2 in the whole range, except some intermediate range. The values of |*Z*_4_| for ST1 and ST2 are magnified by multiplying by a factor of 10^3^ and NS1, NS2, TS1, and TS2 by a factor of 10^2^ and TCS1 and TCS2 are magnified by a factor of 10.

Figure 20 depicts that the behavior of variation of |*Z*_5_| for all the boundary stiffnesses is similar to that of |*Z*_4_|, but the magnitude of variation is different. The values of |*Z*_5_| for ST1, ST2 are magnified by multiplying by a factor of 10^3^ and NS1, NS2, TCS1, TCS2, TS1, and TS2 are magnified by a factor of 10^2^.

From Figure 21, it is noted that the values of |*Z*_6_| for ST1 and ST2 decrease in the range 0° < *θ*_0_ < 45°and then increase with increase in angle of incidence. The values of amplitude ratio for transverse force stiffness and thermal contact conductance decrease in the whole range, except the ranges 16° < *θ*_0_ < 24° and 17° < *θ*_0_ < 23°, respectively. The amplitude ratio for normal force stiffness attains maximum value at normal incidence. The values of |*Z*_6_| for ST1, ST2 are magnified by multiplying by a factor of 10^3^ and NS1, NS2, TCS1, TCS2, TS1, and TS2 are magnified by a factor of 10^2^.

Figure 22 depicts that the values of |*Z*_7_| for all the boundary stiffnesses attain maximum value at the normal incidence and then decrease with oscillation to attain minimum value at the grazing incidence. The values of |*Z*_7_| for ST1 and ST2 are magnified by multiplying by a factor of 10^8^ and NS1, NS2, TS1, TS2, TCS1, and TCS2 are magnified by a factor of 10^6^.

## 9. Conclusion

Reflection and transmission at an interface between heat conducting elastic solid and micropolar fluid media are discussed in the present paper. Effect of normal force stiffness, transverse force stiffness, thermal contact conductance, and thermal relaxation times is observed on the amplitude ratios for incidence of various plane waves (P-wave, T-wave, and SV-wave). When P-wave is incident, it is noticed that the values of amplitude ratio for transverse force stiffness for transmitted T-wave are greater than all the other boundary stiffnesses. When plane wave (SV-wave) is incident, the trend of variation of amplitude ratio for transmitted transverse wave coupled with transverse microrotational wave, that is, C-I and C-II waves, is similar, but magnitude of oscillation is different. The values of amplitude ratio of transmitted LD-wave and T-wave for L-S theory are greater than the value for G-L theory (when T-wave is incident). The model considered is one of the more realistic forms of earth models and it may be of interest for experimental seismologists in exploration of valuable materials such as minerals and crystal metals.

## Appendix

Consider the following:

(A.1) a1i=−c1Knω2Vi1−Vi2V02sin2θ0,  a13=−c1Knω2V0sinθ0,a14=−{(1−V12¯V02sin2θ0)[d2f+d3f(1−V12¯V02sin2θ0)]ω2V12¯    +d1fff1¯+g1¯d1f    +ιc1KnωV1¯(1−V12¯V02sin2θ0)},a15=−{(1−V22¯V02sin2θ0)[d2f+d3f(1−V22¯V02sin2θ0)]ω2V22¯    +d1fff1¯+g2¯d1f    +ιc1KnωV2¯(1−V22¯V02sin2θ0)},a16=d3fω2V3¯V0sinθ01−V32¯V02sin2θ0+ιc1KnωV0sinθ0,a17=d3fω2V4¯V0sinθ01−V42¯V02sin2θ0+ιc1KnωV0sinθ0,a2i=−c2Ktω2V0sinθ0,  a23=c2Ktω2V31−V32V02sin2θ0,a24=(2d4f+d5f)ω2V1¯V0sinθ01−V12¯V02sin2θ0+ιc2KtωV0sinθ0,a25=(2d4f+d5f)ω2V2¯V0sinθ01−V22¯V02sin2θ0+ιc2KtωV0sinθ0,a26=d4fω2V32¯(1−2V32¯V02sin2θ0)+d5f[1−V32¯V02sin2θ0−f3¯] +ιc2KtωV3¯1−V32¯V02sin2θ0,a27=d4fω2V42¯(1−2V42¯V02sin2θ0)+d5f[1−V42¯V02sin2θ0−f4¯] +ιc2KtωV4¯1−V42¯V02sin2θ0,a3i=c4Kθfi,  a33=0,a34=[ιωV1¯1−V12¯V02sin2θ0−c4Kθ]f1¯,a35=[ιωV2¯1−V22¯V02sin2θ0−c4Kθ]f2¯,  a36=a37=0,a4i=(d1+d2(1−Vi2V02sin2θ0))ω2Vi2+(1−τ1ιω)fi,a43=d2ω2V3V0sinθ01−V32V02sin2θ0,a44=−[(d2f+d3f(1−V12¯V02sin2θ0))ω2V12¯+(d1fff1¯+d1fg1¯)],a45=−[(d2f+d3f(1−V22¯V02sin2θ0))ω2V22¯+(d1fff1¯+d1fg1¯)],a46=d3fω2V3¯V0sinθ01−V32¯V02sin2θ0,a47=d3fω2V4¯V0sinθ01−V42¯V02sin2θ0,a51=−(2d4)ω2V1V0sinθ01−V12V02sin2θ0,a52=−(2d4)ω2V2V0sinθ01−V22V02sin2θ0,a53=d4ω2V32(1−2V32V02sin2θ0),a54=−(2d4f+d5f)ω2V1¯V0sinθ01−V12¯V02sin2θ0,a55=−(2d4f+d5f)ω2V2¯V0sinθ01−V22¯V02sin2θ0,a56=−[d5f(ω2V32¯(1−V32¯V02sin2θ0)−f3¯)d4fω2V32¯(1−2V32¯V02sin2θ0)   +d5f(ω2V32¯(1−V32¯V02sin2θ0)−f3¯)],a57=−[(ω2V42¯(1−V42¯V02sin2θ0)−f4¯)d4fω2V42¯(1−2V42¯V02sin2θ0)    +d5f(ω2V42¯(1−V42¯V02sin2θ0)−f4¯)],a6i=a63=a64=a65=0,a66=ιωV3¯f3¯1−V32¯V02sin2θ0,a67=ιωV4¯f4¯1−V42¯V02sin2θ0,a7i=ιωVi1−Vi2V02sin2θ0fi,  a73=0,a74=ιp2ωV4¯1−V12¯V02sin2θ0f1¯,a75=ιp2ωV5¯1−V22¯V02sin2θ0f2¯,a76=a77=0, (i=1,2),d1=λρc12,  d2=2μρc12,  d3=d22,d1ff=bν,  d1f=c0ρνT0,  d2f=λfω∗ρc12,d3f=(2μf+Kf)ω∗ρc12,  d4f=μfω∗ρc12,d5f=Kfω∗ρc12,  p2=K1∗K∗,c1=c2=νT0ρc12,  c4=νc12ω∗K1∗.

## Symbols

*λ*, *μ*: Lame's constants
*t*_*ij*_: Components of the stress tensor
$\vec{u}$: Displacement vector
*ρ*: Density
*K*^*^: Thermal conductivity
*c*^*^: Specific heat at constant strain
*T*_0_: Uniform temperature
*T*: Temperature change
*α*_*T*_: Coefficient of linear thermal expansion
*δ*_*ij*_: Kronecker delta
*ε*_*ijr*_: Alternating symbol
*λ*^*f*^, *μ*^*f*^, *K*^*f*^,
*α*^*f*^, *β*^*f*^, *γ*^*f*^, *c*_0_: Material constants of the fluid
*σ*_*ij*_^*f*^: Components of stress tensor in the fluid
*m*_*ij*_^*f*^: Components of couple stress tensor in the fluid
$\vec{v}$: Velocity vector
$\vec{\Psi }$: Microrotation velocity vector
*ρ*^*f*^: Density
*I*: Scalar constant with the dimension of moment of inertia of unit mass
*p*: Pressure
*K*_1_^*^: Thermal conductivity
*aT*_0_^*f*^: Specific heat at constant strain
*T*_0_^*f*^: Absolute temperature
*T*^*f*^: Temperature change
*ϕ*^∗*f*^: Variation in specific volume
*α*_*T*^*f*^_: Coefficient of linear thermal expansion.
